# Serum uric acid and the incidence of new-onset metabolic syndrome among military young adults in Taiwan: the CHIEF cohort study

**DOI:** 10.3389/fcdhc.2026.1822830

**Published:** 2026-04-30

**Authors:** Wei-Che Huang, Yun-Chen Chang, Kun-Zhe Tsai, Chen-Ming Huang, Han-Hsing Chen, Hui-Shan Wang, Gen-Min Lin

**Affiliations:** 1 Division of Cardiovascular Surgery, Department of Surgery, Tri-Service General Hospital, National Defense Medical University, Taipei, Taiwan; 2 Nursing Department, China Medical University Hospital, Taichung, Taiwan; 3 School of Nursing and Graduate Institute of Nursing, China Medical University, Taichung, Taiwan; 4 Department of Stomatology of Periodontology, Mackay Memorial Hospital, Taipei, Taiwan; 5 Divison of Cardiovascular Surgery, Department of Surgery, Mennonite Christian Hospital, Hualien, Taiwan; 6 Division of Cardiology, Department of Internal Medicine, Mennonite Christian Hospital, Hualien, Taiwan; 7 Division of Cardiology, Department of Internal Medicine, Hualien Tzu Chi General Hospital, Hualien, Taiwan; 8 Department of Internal Medicine, Tri-Service General Hospital, National Defense Medical University, Taipei, Taiwan; 9 Department of Medicine, Hualien Armed Forces General Hospital, Hualien, Taiwan

**Keywords:** cohort study, hyperuricemia, metabolic syndrome, serum uric acid, young adults

## Abstract

**Backgrounds:**

Serum uric acid (SUA), a purine metabolite, can reflect the status of low-grade inflammation in the body, which may contribute to metabolic syndrome (MetS). However, the association has been found to be weak in men, especially in elderly individuals. Whether this association is present in young men remains unclear. This study aimed to investigate the association between SUA concentrations and the incident MetS among military personnel, most of whom were young men.

**Materials and methods:**

This cohort study included 2,890 Taiwanese military personnel (90% male), aged 18–39 years, who were free of MetS at baseline following new-onset MetS from 2014 (baseline) through the end of 2020. SUA concentrations were measured at baseline. Incident MetS was diagnosed using the modified NCEP ATP III criteria and was identified during the annual military health examinations. A multivariable Cox regression analysis model adjusted for baseline age, sex, substance use, physical activity level, body mass index, blood urea nitrogen level, serum creatinine level, total white blood cell count and hemoglobin level was used to determine the association. Subgroup analyses were performed for each component of MetS.

**Results:**

During a mean follow-up of 6.0 years, 673 incident MetS events (23.3%) were reported. Higher SUA concentrations (every 1 mg/dL increase) were associated with a greater risk of MetS [hazard ratio: 1.22 (95% confidence interval: 1.15–1.30)]. There were no significant differences within each MetS component, i.e., central obesity, hypertension, hyperglycemia or dyslipidemia, according to the subgroup analyses.

**Conclusion:**

This study suggests that higher SUA concentrations are associated with a greater risk of new-onset MetS among Taiwanese military personnel, most of whom were young men, and the association was consistent across the MetS subgroups. Since this prospective cohort study is observational, the cause-and- effect association requires further investigation.

## Introduction

Metabolic syndrome (MetS) is a medical condition characterized by the presence of a group of cardiovascular disease (CVD) risk factors, including insulin resistance, abnormal blood lipids (dyslipidemia), central obesity, and hypertension (1, 2). The prevalence of MetS is approximately 26.4% in the Chinese adult population according to the International Diabetes Federation (IDF) cirteria (3). MetS plays a critical role in mediating the development of chronic kidney disease (CKD) and CVD, namely, CVD-CKD-MetS (CKM) syndrome, which threatens public health in the modern world (4, 5). The prevalence of MetS increases with age (6), particularly from young to middle age. The prevalence of MetS in a specific population of young military adults in Taiwan, aged 18–39 years with an average age of 28 years, was found to be approximately 11%, which increased threefold to 34% after a 6-year follow-up (7–9). Therefore, it is crucial to identify early markers of MetS and prevent its development before middle age in developing and developed countries.

Uric acid can serve as a protective antioxidant in hydrophilic environments by scavenging specific oxidants, known as a complex redox paradox (10). Serum uric acid (SUA) concentrations increase mainly because of the ingestion of a high-purine diet or reduced discharge from the kidneys. Hyperuricemia (11) is associated with not only gouty arthritis (12) but also CKD (13) and MetS (14). In addition, higher baseline SUA levels and the presence of subcutaneous tophi have been observed to be independent predictors of all-cause mortality in individuals with gout, with the majority of excess deaths secondary to CVD events (15). It is obvious that SUA can be a crucial mediator in CKM syndrome. However, most studies have been carried out in middle-aged and old-aged individuals, and only a few were in young adults (16, 17). In addition, the results may vary across populations. For instance, the association has been found to be stronger in women than in men (18–20). Therefore, this study aimed to investigate the association between SUA levels and incident MetS in a large cohort of Taiwanese military personnel who were predominantly male.

## Methods

### Study population

The cohort included 4,080 male and female military personnel aged 18–50 years from the Cardiorespiratory Fitness and Health in Eastern Armed Forces (CHIEF) study in the Hualien and Taitung Counties in Taiwan in 2014 (21–23). The participants did not take antihypertensive, antidiabetic, lipid-lowering or urate-lowering medications. Each participant underwent anthropometric, hemodynamic and blood biomarker measurements during annual health examinations. In addition, current substance use status, i.e., betel nut chewing, alcohol intake and cigarette smoking (active vs. former or never), and moderate-intensity physical activity, which was evaluated by weekly running hours (<150 minutes (mins)/week (wk), 150–299 mins/wk and ≥300 mins/wk) (24), during the past six months, were self-reported at baseline (25, 26).

### Annual health examinations (2014–2020)

#### Anthropometric measurements

All participants’ body height, body weight and waist circumference (WC) were measured while in the standing position. Body mass index (BMI) was defined as body weight (kg) divided by body height squared (m (2)). Participants who were classified in the obesity/overweight subgroup were defined as having a BMI ≥25 kg/m^2^ on the basis of the World Health Organization (WHO) criteria for the general population (27, 28).

#### Hemodynamic measurements

The blood pressure (BP) of each participant was measured once via the right arm in the sitting position by the oscillometric method through an automatic BP device (FT201 Parama-Tech Co., Ltd., Fukuoka, Japan) after an adequate duration of rest longer than 15 minutes (29–31). If the initial resting BP was ≥130/80 mmHg, a second BP measurement was obtained after a 15-minute rest period. The final BP level was obtained using an average of the initial BP level and the second BP level.

#### SUA, MetS biomarker and covariate measurements

Serum concentrations of total cholesterol, high-density lipoprotein cholesterol (HDL-C), triglycerides, fasting glucose, SUA, blood urea nitrogen (BUN) and creatinine were measured from an overnight 12-hour fasting blood sample of each participant through an autoanalyzer (Olympus AU640, Kobe, Japan). Hyperuricemia was defined as an SUA concentration ≥7.0 mg/dL in men and ≥6.0 mg/dL in women (32). White blood cell (WBC) counts (33) and hemoglobin levels (34) were measured using an automated hematology analyzer (Sysmex XT- 2000-I, Sysmex America, IL, USA).

### Definition of incident MetS events

Incident MetS was diagnosed according to the National Cholesterol Education Program Adult Treatment Panel III (NCEPATP III) criteria for Chinese adults (35) as the presence of three or more of the following clinical characteristics: (1) HDL-C <40 mg/dL for men and <50 mg/dL for women; (2) serum triglycerides ≥150 mg/dL or with lipid-lowering medications; (3) fasting plasma glucose ≥100 mg/dL or with antidiabetic medications; (4) WC ≥90 cm for men and ≥80 cm for women; and (5) systolic BP ≥130 mmHg or diastolic BP ≥85 mmHg or with antihypertensive therapy.

### Statistical analysis

The baseline characteristics of the participants are presented as the mean ± standard deviation and numbers (%) for continuous variables and categorical variables, respectively. The follow-up of each participant began at baseline (2014) and continued until the first development of MetS events, loss to follow-up and at the end of follow-up (December 31, 2020). SUA concentrations were grouped by quartiles, and a Kaplan–Meier curve was used for survival analysis (free of incident MetS), which was compared by the log-rank test. Multivariate Cox proportional hazards regression analysis was used to determine the association of SUA concentration (treated as a continuous variable per 1 mg/dL increase) with incident MetS events, with simultaneous adjustments for baseline age, sex, smoking status, alcohol intake status, betel nut chewing status, physical activity level and BMI (Model 1). BUN and serum creatinine levels were additionally adjusted in Model 2. WBC and blood hemoglobin levels were further adjusted in Model 3. The potential covariates were selected in the models mainly on the basis of their associations with SUA concentration and MetS in previous studies (7–9). Stratified analyses exploring the associations between SUA concentration and the incidence of MetS according to sex (males vs. females), BMI status (with vs. without obesity/overweight), BP status (with vs. without hypertension), fasting plasma glucose status (with vs. without hyperglycemia) and triglyceride status (with vs. without hypertriglyceridemia), and formal testing for multiplicative interactions were performed. All the statistical analyses were performed using SPSS v25.0 software for Windows (IBM Corp., Armonk, NY, USA). This cohort study was performed in compliance with the principles of the Declaration of Helsinki. In addition, the study design and protocol were reviewed and approved by the Institutional Review Board (IRB) of the Mennonite Christian Hospital (certificate No. 16-05-008) in Hualien City, Taiwan. Written informed consent was obtained from all the subjects.

## Results

In this study, participants who had baseline MetS (N = 457) and a baseline age ≥40 years (N = 58) and those who moved out of the military bases in Eastern Taiwan and who were lost to follow-up (N = 675) were excluded, resulting in a final sample of 2,890 participants who have complete data for analysis.

The clinical characteristics of the participants with and without hyperuricemia are shown in **Table 1**. Compared with those without hyperuricemia, more participants with hyperuricemia (n =925) were male (97.9% vs. 85.2%). Those with hyperuricemia had more metabolic problems, such as higher BMI, WC, systolic BP, diastolic BP, and serum triglyceride levels, but, conversely, had lower HDL-C levels. In addition, those with hyperuricemia had greater BUN levels, serum creatinine levels, WBC counts and hemoglobin levels. The prevalence of alcohol intake and betel nut chewing was significantly greater in those with hyperuricemia, whereas physical activity levels and the prevalence of cigarette smoking did not differ significantly between the two groups.

**Table 1:** Baseline characteristics of the military cohort with and without hyperuricemia (N =2,890).

Variables	Without hyperuricemia (N =1,965)	With hyperuricemia (N =925)	p-value
Plasma metabolic biomarkers			
Serum uric acid, mg/dL	5.76 ± 0.89	7.99 ± 0.89	<0.001
HDL-C, mg/dL	50.83 ± 10.11	48.01 ± 9.54	<0.001
Serum triglycerides, mg/dL	91.33 ± 51.21	108.83 ± 70.71	<0.001
Fasting glucose, mg/dL	91.72 ± 9.72	92.58 ± 9.74	0.02
Age, years	28.29 ± 5.78	28.57 ± 5.82	0.22
Sex, %			
Men	1,675 (85.2)	906 (97.9)	<0.001
Women	290 (14.8)	19 (2.1)	
BMI, kg/m^2^	23.75 ± 2.87	25.37 ± 2.96	<0.001
Waist circumference, cm	79.97 ± 7.86	84.57 ± 7.57	<0.001
Systolic BP, mmHg	114.91 ± 12.80	117.97 ± 12.86	<0.001
Diastolic BP, mmHg	68.64 ± 9.39	70.69 ± 10.03	<0.001
Lifestyle behavior, %			
Alcohol drinking	733 (37.3)	428 (46.3)	<0.001
Betel nut chewing	155 (8.0)	124 (13.5)	<0.001
Cigarette smoking	671 (34.6)	320 (34.9)	0.84
Physical activity level, %			
<150 min/wk	411 (22.4)	186 (20.1)	0.21
150 – 299 min/wk	755 (38.4)	349 (37.7)	
≥300 min/wk	769 (39.1)	390 (42.2)	
White blood cell count, 10^3^/μL	6.52 ± 1.62	6.86 ± 1.65	<0.001
Hemoglobin, g/dL	14.80 ± 1.22	15.15 ± 1.00	<0.001
BUN, mg/dL	12.34 ± 2.84	13.34 ± 2.93	<0.001
Serum creatinine, mg/dL	0.90 ± 0.13	0.99 ± 0.12	<0.001
Metabolic syndrome event, %	347 (17.7)	326 (35.2)	<0.001

Data are presented as mean (standard deviation) and numbers (percentage).

Definition: hyperuricemia is defined as serum urate ≥7.0 mg/dL in men and ≥6.0 mg/dL in women. BMI, body mass index; BP, blood pressure; BUN, blood urea nitrogen; HDL-C, high-density lipoprotein cholesterol.

**Figure 1:**
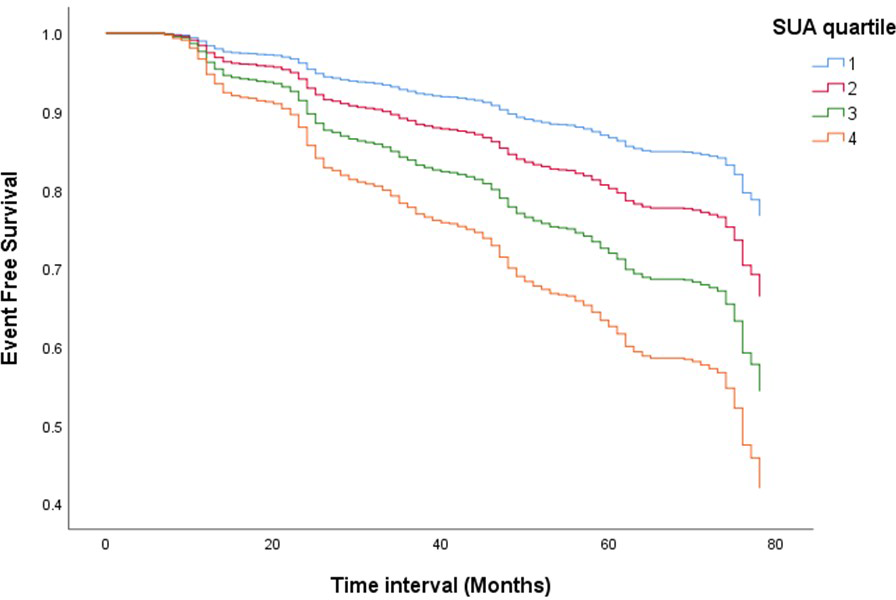
Kaplan–Meier survival curves for the cumulative probability of MetS event-free survival across the four SUA quartiles over a 6-year follow-up period. The survival curves for the four groups began to diverge early in the follow-up period and continued to separate progressively over time. Participants in the highest SUA quartile (Q4) experienced the greatest decline in event-free survival, which revealed the highest cumulative incidence of MetS. The survival distributions differed significantly among the four groups (p < 0.001).

During a mean follow-up of 6.0 years, 673 incident MetS events (23.3%) were reported [326 (35.2%) and 347 (17.7%) in those with and without hyperuricemia, respectively]. **Figure 1** shows the Kaplan-Meier survival curves for the cumulative probability of MetS event-free survival across the four SUA quartiles over a 6-year follow-up period. The survival curves for the four groups began to diverge early in the follow-up period and continued to separate progressively over time. Participants in the highest SUA quartile (Q4) experienced the greatest decline in event-free survival, which revealed the highest cumulative incidence of MetS. The survival distributions differed significantly among the four groups (p <0.001).

As shown in **Table 2**, for the multivariable Cox regression analysis results, the unadjusted model revealed a significant association between SUA concentration and incident MetS (hazard ratio (HR): 1.41 [95% confidence interval (CI)]: 1.34–1.48). After adjusting for the covariates in Models 1, 2 and 3, the associations remained statistically significant [HRs: 1.22 (95% CI: 1.15–1.29) and 1.24 (95% CI: 1.17–1.32) and 1.22 (95% CI: 1.15–1.30)].

**Table 2:** Multivariable cox regression analysis for incidence of metabolic syndrome with serum uric acid.

Variables		Crude model				Model 1					Model 2				Model 3	
	HR	95% CI	p		HR	95% CI	p			HR	95% CI	p		HR	95% CI	p
Serum uric acid	1.41	1.34 – 1.48	<0.001		1.22	1.15 – 1.29	<0.001			1.24	1.17 – 1.32	<0.001		1.22	1.15 – 1.30	<0.001

Data are presented as hazard ratio (HR) and 95% confidence interval (CI) using multivariable Cox regression analysis for Model 1: age, sex, alcohol drinking, betel nut chewing, cigarette smoking, physical activity and body mass index adjustments; Model 2: Model 1 + blood urea nitrogen and serum creatinine adjustments.

Model 3: Model 2 + white blood cell count and hemoglobin adjustments.

**Table 3** shows the subgroup analyses for the association of SUA concentration with incident MetS. The association was consistent across all the subgroups stratified by the MetS components, and there were no statistically significant differences within each MetS component.

**Table 3:** Association of serum uric acid with incident of metabolic syndrome among young adults in subgroup analyses.

Baseline	N	HR	95% CI	p-value
Waist circumference				
With central obesity	502	1.20	1.08 – 1.34	0.001
Without central obesity	2,388	1.23	1.14 – 1.33	<0.001
BP				
With hypertension	456	1.18	1.03 – 1.35	0.01
Without hypertension	2,434	1.23	1.14 – 1.32	<0.001
HDL-C				
Under lower limit	433	1.21	0.97 – 1.28	0.09
Over lower limit	2,457	1.23	1.13 – 1.30	<0.001
Serum triglycerides				
≥150 mg/dL	337	1.19	1.05 – 1.34	0.004
<150 mg/dL	2,553	1.23	1.13 – 1.31	<0.001
Fasting glucose				
≥100 mg/dL	360	1.25	1.07 – 1.46	0.004
<100 mg/dL	2,530	1.21	1.16 – 1.32	<0.001

Data are presented as hazard ratio (HR) and 95% confidence interval (CI) using multivariable Cox regression analysis for age, sex, alcohol drinking, betel nut chewing, cigarette smoking, physical activity, body mass index, blood urea nitrogen, serum creatinine, white blood cell count and hemoglobin adjustments.

Definitions: central obesity is defined as waist circumference ≥90 cm in men and ≥80 cm in women; the lower limit of HDL-C is defined as 40 mg/dL in men and 50 mg/dL in women. BP, blood pressure; HDL-C, high-density lipoprotein cholesterol.

## Discussion

Our main findings were that SUA concentration is significantly positively associated with new-onset MetS development among young Taiwanese adults, most of whom were young men, even adjusting for multiple traditional risk factors, physical activity, BMI, kidney function, inflammation markers and hemoglobin levels. In addition, the association was consistent regardless of the status of the metabolic disorders presented.

Previous studies have shown that hyperuricemia is associated with a greater risk of MetS. Some cohort studies have reported greater associations in women than in men (18–20). In older populations, the association was present in women but not in men (20). Since age and sex are crucial modifiers of the association between SUA concentration and new-onset MetS, this prospective cohort study confirmed that a positive association exists among young military adults in Taiwan, 90% of whom were male. To the best of our knowledge, in older individuals, SUA concentrations might be affected by many comorbidities, such as CKD, which can reduce SUA discharge, leading to an overestimation of oral food intake. In other respects, older individuals may have multiple comorbidities, which are likely to lead to cachexia and thus reduce waist size and body weight (36–38).

Kuwabara et al. demonstrated that hyperuricemia is associated with a higher cumulative incidence of dyslipidemia, hypertension, obesity/overweight and CKD in a large sample of Japanese individuals without baseline comorbidities during a 5-year period (39). Our findings demonstrated that in the absence of comorbidities, particularly dyslipidemia, hypertension and central obesity, the risk of new-onset MetS with greater SUA concentration seemed to be higher despite the associations not being significantly different within each subgroup. This condition may be caused by individuals with obesity or dyslipidemia who have a greater risk of MetS development and are associated with insulin resistance and unhealthy dietary patterns, possibly reducing the contribution of SUA. Conversely, in metabolically healthy individuals, elevated SUA concentrations may serve as a sensitive, early biomarker of metabolic derangements such as fructose overconsumption (40), incipient insulin resistance (41), or subclinical inflammation (42, 43) that has not yet been presented as established obesity. Therefore, higher SUA could help to identify the potential risk of MetS progression in relatively healthy groups. Mechanisms for the increased risk of MetS development have been proposed: hyperuricemia is related to mitochondrial dysfunction (44) and stimulates proinflammatory pathways (44–46), thereby promoting insulin resistance and hepatic lipogenesis even without excessive caloric intake (47, 48). Furthermore, uric acid is a potent inducer of oxidative stress which can cause endothelial dysfunction (49, 50). These mechanisms support our findings that SUA concentration is intrinsically linked to the pathogenesis of metabolic components.

### Study strengths and limitations

This study has several strengths and limitations. First, the sample size of this study cohort was large, with a longitudinal design. Second, the demographic characteristics in this study included a homogenous, young military cohort, minimizing the confounds of age-related comorbidities and interference from medications, which is often seen in older populations. Third, all the examinations were carried out at the same institution, which may avoid many technical biases. In contrast, military personnel are highly specific that would restrict generalizability, particularly to women and community-based civilian populations.

## Conclusion

This study suggests that higher SUA concentrations are associated with a greater risk of new-onset MetS among Taiwanese military personnel, most of whom were young men, and the association was consistent across MetS subgroups.

## Data Availability

The raw data supporting the conclusions of this article will be made available by the authors, without undue reservation.
